# Recognizing the Early Risk-Based Clinical Manifestations of Mucormycosis: Cornerstones for Improved Survival and Therapeutic Outcomes

**DOI:** 10.3390/jof11070507

**Published:** 2025-07-04

**Authors:** Panagiotis Zagaliotis, Thomas J. Walsh

**Affiliations:** 1 Division of Infectious Diseases, Weill Cornell Medicine, New York, NY 10065, USA; paz4002@med.cornell.edu; 2 Center for Innovative Therapeutics and Diagnostics, Richmond, VA 23220, USA; 3 Departments of Medicine and Microbiology & Immunology, University of Maryland School of Medicine, Baltimore, MD 21201, USA

## 1. Introduction

The early diagnosis and treatment of mucormycosis improves outcomes and saves lives. Critical to establishing an early diagnosis are the awareness of high-risk patient populations and the clinical recognition of the early manifestations of mucormycosis at specific anatomic sites. Although previous publications have emphasized the importance of early diagnosis, the clinical manifestations that are discussed represent classical advanced stages of infection. This review will provide a foundation for understanding high-risk patient populations and the early clinical manifestations of mucormycosis that may lead to rapid implantation of diagnostic procedures and therapeutic interventions.

Mucormycosis has emerged as an increasingly important cause of morbidity and mortality among immunocompromised patients [1,2,3,4]. The diseases caused by *Rhizopus arrhizus* and other specific members of the order Mucorales involve the lungs, sinuses, brain, and other tissue sites with angioinvasion, leading to infarction and necrosis.

The majority of cases of mucormycosis in humans are caused by members of the order Mucorales. Organisms of the genus *Rhizopus* are the most common clinical isolates, with *R. arrhizus* being the most frequently recovered species. The next species in order of frequency are *Mucor* spp. and *Lichtheimia* (formerly *Absidia*) spp., while *Cunninghamella, Apophysomyces, Saksenaea, Rhizomucor*, and other genera each represent a significantly smaller percentage of clinical isolates.

## 2. Epidemiology

Human respiratory tract infection is acquired through the inhalation of sporangiospores from environmental sources. The acquisition of hyphae and sporangiospores from environmental sources via the cutaneous or percutaneous route is also common, either through the disruption of skin barriers in trauma victims or from the invasion of macerated skin in transplant recipients. Infection through the gastrointestinal route also may occur, especially among premature neonates [5].

The estimated incidence of mucormycosis was 1.7 cases per million/year in a population-based study performed in the San Francisco Bay area [6]. While the low incidence and prevalence of mucormycosis warrant its designation as a rare disease, it is considerably more common in high-risk patients. For example, the incidence of mucormycosis-related hospitalizations in US hospitals was estimated at 1.2 per 100,000 discharges between 2005 and 2014 [7]. The incidence of mucormycosis also may be increasing. The annual incidence of mucormycosis in a university hospital of Belgium increased from 0.019 cases/10,000 patient-days in 2000 to 0.148 cases/10,000 patient-days in 2009 [8]. This increased incidence may be attributed to better diagnostic methods, increased awareness, as well as an expansion of populations at risk for mucormycosis. By maintaining a high index of suspicion of the initial clinical manifestations of mucormycosis in high-risk patients, mucormycosis can be treated in its early stages.

## 3. Recognition of High-Risk Patients

In comparison to other opportunistic mycoses, mucormycosis occurs in a heterogeneous host population. Diabetes mellitus (DM), both type I and type II, is the most common underlying condition for development of mucormycosis. Ketoacidosis is an important risk factor in DM. Metabolic acidosis secondary to other causes, such as primary aminoaciduria, also increase the risk for mucormycosis [5].

Hematological malignancies, severe aplastic anemia, myelodysplasia, HCT, and SOT are important underlying conditions for the development of mucormycosis. Prolonged neutropenia, corticosteroid use, graft versus host disease (GvHD), and qualitative defects in phagocytic function contribute to the risk of mucormycosis in patients with acute leukemia and HCT. Musculoskeletal trauma caused by explosives, storms, burns, and illicit intravenous drug use (Table 1). Further increasing the need for awareness of mucormycosis, there have been significant increases in the incidence of mucormycosis during the past three decades among patients with acute leukemia, HCT, SOT, and illicit IV drug use [1,9,10].

An increasing number of cases of mucormycosis have also been reported in previously immunocompetent patients in the settings of penetrating trauma, combat blast injury, and burns [11,12]. Penetrating injuries that patients sustain from tornadoes and tsunamis have been complicated by myocutaneous and musculoskeletal mucormycosis. Thus, clinicians should consider mucormycosis within the differential diagnosis of severe, necrotizing, and non-healing wounds of patients suffering from trauma related to combat blast injuries, burns automobile injuries, industrial accidents, tornadoes, and tsunamis.

COVID-19, particularly in patients with DM who have received corticosteroids and other immunosuppressive agents for the inflammatory responses to the virus, constitute a relatively new risk group [13,14,15]. The convergence of poorly controlled DM, development of COVID-19 inflammatory pulmonary infiltrates, and the systemic immunosuppressive effects of corticosteroids markedly increases the risk of development of sino-orbital and rhinocerebral mucormycosis.

## 4. Host Defenses and Pathogenesis

The impaired host defenses encountered in DM, hematological malignancies, HCT, SOT, and other hosts increase the risk for mucormycosis. A detailed description of host defenses and the pathogenesis of mucormycosis is beyond the scope of this manuscript. Quantitative and qualitative defects in polymorphonuclear leukocytes (PMNs) and pulmonary alveolar macrophages (PAMs) impair phagocytosis of sporangiospores and damage to hyphae [16,17,18]. Additional defects in metabolic acidosis, iron sequestration, and increased expression of glucose-related protein 78 (GRP-78) in DM further increase the risk of mucormycosis in this vulnerable patient population [19]. These impairments of host defenses define the patient population at risk in whom the early clinical manifestations of mucormycosis are imperative to recognize.

The clinical manifestations of mucormycosis are principally a reflection of its destructive angioinvasive properties [16,18,20,21]. Angioinvasion leads to thrombosis, ischemia, and infarction of neurovascular tissues of the orbit and brain. Within the lung, angioinvasion results in hemorrhagic infarcts and the reversed halo sign, while within the skin and subcutaneous tissues, it causes non-healing eschar-like lesions and deep soft tissue necrosis.

## 5. Recognition of Early Clinical Manifestations

The clinical manifestations of mucormycosis are classified according to the following anatomic patterns: paranasal sinus infection, sino-orbital disease, rhinocerebral infection, pneumonia, primary cutaneous disease, musculoskeletal infection, gastrointestinal disease, single organ disease, and disseminated mucormycosis [1,22,23,24]. There is a host-based propensity for certain patterns of mucormycosis to be associated with specific host groups (Table 2). For example, while sino-orbital mucormycosis is the most common clinical manifestation in patients with DM, pulmonary disease is uncommon. By comparison, sino-pulmonary disease, especially of the lung, is the most common pattern observed in patients with hematological malignancies.

The early clinical manifestations of mucormycosis have a relatively broad differential diagnosis in a general patient population that encompasses microbiological causes and non-microbiological etiologies, including bacterial infection, insect bites, angioedema, orbital cellulitis, Pott’s puffy tumor, chalazion, and orbital pseudotumor. However, within the context of high-risk and immunocompromised patients, such as those with diabetes mellitus or neutropenia, the differential diagnosis is narrowed to include a high probability of sino-orbital mucormycosis. Table 3 provides a guide to recognition of the early clinical manifestation of mucormycosis in the context of high-risk patients.

### 5.1. Paranasal Sinus, Sino-Orbital, and Rhinocerebral Mucormycosis

Mucormycosis of the paranasal sinuses may be restricted to the sinuses or may invade the orbit (sino-orbital mucormycosis). Further extension from the paranasal sinuses into the brain tissue may ensue (rhinocerebral mucormycosis). Mucormycosis develops in the paranasal sinuses of high-risk patients following an inhalation of sporangiospores.

While initial clinical manifestations of paranasal sinus, sino-orbital, and rhinocerebral mucormycosis are relatively non-specific in the general medical and pediatric population, they represent early sentinels of this fungal disease that should prompt a rapid evaluation and therapeutic intervention, including empirical antifungal therapy, surgical consultation, and endoscopic evaluation. Nasal endoscopy typically reveals the sino-nasal mucosa to become red, violaceous, and finally black, as angioinvasion leads to tissue necrosis. Endoscopy may reveal blackened necrotic crusts on the nasal septum and turbinates. These “sentinel eschars” may represent an early phase of infection and may be more amenable to biopsy than maxillary, ethmoidal, sphenoidal, or frontal sinus lesions. Biopsied tissue is submitted for direct examination, cultures, and histology. Antifungal therapy is empirically initiated and the patient further evaluated for surgery if there is microbiological evidence of mucormycosis.

### 5.2. Mucormycosis of the Maxillary Sinuses

Mucormycosis of the maxillary sinuses may present early with facial erythema, pain, and local tenderness. A blood-tinged or black nasal discharge may be apparent. A local maxillary sinus infection may also present with local pain in the roots of the first and second premolars [25]. Maxillary sinus mucormycosis may be detected at an early stage by recognition of an ipsilateral erythema or subtle violaceous hue of the hard palate as the result of invasion of the greater palatine artery (a branch of the maxillary artery). Alternatively, a more advanced stage of hard-palate infection may be evident by a painful focal palatal necrosis as the result of invasion of the floor of the maxillary sinus.

### 5.3. Mucormycosis of the Ethmoidal Sinuses

Due to its close proximity to the orbits, eyes, and critical veinous drainage to the cavernous sinuses, mucormycosis of the ethmoid sinuses represents a medical and surgical emergency. Early detection and treatment of mucormycosis of the ethmoid sinuses may be sight-saving and prevent sino-orbital and cerebral infection.

Periorbital cellulitis is a common early manifestation of mucormycosis of the ethmoid sinuses. Periorbital cellulitis, which may be accompanied by lacrimation and chemosis, in the aforementioned high-risk patient populations should prompt an evaluation for ethmoid sinus infection of which the differential diagnosis includes ethmoidal mucormycosis. The evaluation of periorbital cellulitis in the high-risk patient should include CT scan of the sinuses and otolaryngological consultation, including assessment for nasal endoscopy. The objective of this early diagnosis is to prevent development of sino-orbital mucormycosis.

### 5.4. Sino-Orbital Mucormycosis

Diplopia in a high-risk patient also may be an early sign of ethmoidal and sino-orbital mucormycosis. Extension of ethmoidal mucormycosis into the orbit occurs through hyphal invasion of the lamina paprycea, which is contiguous with the medial rectus extraocular muscle. Invasion of the medial rectus muscle impairs its extraocular muscle mobility leading to ophthalmoplegia, dysconjugate gaze, and diplopia. For patients with DM, while hypoglycemia may lead to blurry vision caused by alteration of the lens crystalline structure, it does not result in diplopia.

Later stages of ethmoidal and sino-orbital mucormycosis that demonstrate proptosis and a blackened eschar over the ethmoidal area may require more extensive surgery and result in loss of the vision of the involved eye. Ocular or optic nerve involvement is suggested by loss of vision. Posterior extension of the orbital infection may result in the orbital apex syndrome with catastrophic infarction of the neurovascular structures within the optic canal (optic nerve and ophthalmic artery) and superior orbital fissure (cranial nerves III, IV, V1, VI), as well as veins draining the orbit into the cavernous sinus.

### 5.5. Pulmonary Mucormycosis

Pulmonary mucormycosis is most commonly observed in patients with hematological malignancies, as well as HCT and SOT recipients [1,26,27]. Other patient populations with severe aplastic anemia, myelodysplasia, corticosteroid therapy, and deferoxamine treatment are also at risk for pulmonary mucormycosis. Sinus and pulmonary infections occur more frequently in mucormycosis than with aspergillosis [28,29,30,31,32,33].

Patients may manifest early symptoms and signs of fever refractory to broad spectrum antibacterial agents, cough, production of blood-tinged respiratory secretions, and pleuritic pain. While none of these findings alone are indicative of pulmonary mucormycosis, within the context of profound neutropenia and other immunosuppressive therapies, patients should be evaluated by chest and sinus CT scans as promptly as possible.

Early pulmonary disease caused by mucormycosis may be manifested as multifocal nodular or alveolar infiltrates, consolidation, and pleural effusions. As the fungal disease progresses through lung tissue via pulmonary angioinvasion, thrombosis and infarctions result in consolidated masses, intraparenchymal hemorrhage, and hemoptysis, especially with the involvement of pulmonary arteries.

The development of early signs and symptoms of pulmonary mucormycosis, albeit not specific for this disease, should increase the index of suspicion to performing diagnostic procedures, including the evaluation of BAL fluid by direct exam, culture, and PCR, as well as multiplex next-generation sequencing and cell-free targeted PCR.

### 5.6. Cutaneous Mucormycosis

Cutaneous mucormycosis in immunocompromised patients may represent an early manifestation of invasive disease as the portal of entry for disseminated infection [1,34]. These lesions usually have resulted from direct percutaneous inoculation of fungal elements from contaminated sources, especially plants, soil, or other organic materials. A careful history may elicit such traumatic exposure. Early detection and resection of such lesions may prevent dissemination as well as preserve local tissue. Such lesions are characterized as poorly healing ulcerations with indurated surrounding ischemic tissue, and progressive necrotic foci with hardened black eschars.

Alternatively, immunocompetent patients with no apparent underlying conditions may also suffer from cutaneous mucormycosis as a result of direct inoculation for combat blast injury, vehicular trauma, burns, earthquakes, tornadoes, and tsunami [5,11,34,35,36,37,38,39]. Alternatively, it may occur in the context of disseminated disease or extensive local infection in immunocompromised hosts. Prematurely born infants also are highly vulnerable to the development of cutaneous mucormycosis due to the tenuous structure of their skin exposed to sporangiospores on contaminated linens.

Initial lesions may appear as necrotic punctate foci, which expand radially to in cutaneous, subcutaneous, or deep musculoskeletal tissues, depending on the type and depth of injury. Extensive local invasion may occur involving the adjacent subcutaneous fat, muscle, fascia, tendon, and osseous tissues.

Cutaneous mucormycosis may also be the result of disseminated disease, with an initial presentation of nodular erythematous subcutaneous lesions. These lesions may progress to necrotic ulcerations.

Biopsy, wet mounts, and culture of suspected lesions at the earliest possible stage of infection, demonstrating broad ribbon-like aseptate or pauci-septate hyphae with angioinvasion, confirms the clinical diagnosis and allows for appropriate antifungal therapy and surgical interventions [36].

### 5.7. Gastrointestinal Mucormycosis

Gastrointestinal infection is an uncommon form of mucormycosis that occurs in immunocompromised patients, premature neonates, and malnourished patients and may present as necrotizing enterocolitis [5,40]. Following ingestion of sporangiospores, hyphal invasion of the mucosa, submucosa, and vascular structures of the gastrointestinal tract may occur. The resulting necrotic ulcers may lead to a ruptured intestinal wall and peritonitis. The nonspecific symptoms of gastrointestinal mucormycosis, which include fever, abdominal pain, peritoneal signs, distention, and hemorrhage, should prompt urgent gastroenterological and surgical evaluation. An early diagnosis, surgical intervention, and antifungal therapy may be life-saving.

## 6. Recognition of Advanced Clinical Manifestations

Diagnosis of mucormycosis may be delayed in high-risk patients for various reasons, including limited access to health care facilities and lack of clinical recognition of early manifestations. The traditional training in undergraduate medical school curricula and in postgraduate programs usually emphasizes the classic advanced manifestations of mucormycosis. These clinical manifestations of mucormycosis are nevertheless important to recognize, evaluate, and treat as rapidly as possible to improve outcome (Table 4).

### 6.1. Paranasal Sinus, Sino-Orbital, and Rhinocerebral Mucormycosis

#### 6.1.1. Advanced Mucormycosis of the Maxillary Sinuses

Mucormycosis of the maxillary sinuses may extend into the hard palate resulting in necrosis, perforation, and rapid destruction of palatine bones. Infection may also destroy dental roots of the first and second premolars, resulting in tooth loss. Extension into anterolateral maxillofacial soft tissue may result in tissue necrosis of these structures.

#### 6.1.2. Advanced Mucormycosis of the Ethmoidal Sinuses

Infarction of the ethmoid sinus may extend through the lamina paprycea and into the orbit with initial invasion of the medial rectus. This manifestation may still be sufficiently early in its development to allow for rapid ophthalmological and otolaryngological surgical intervention to save the eye.

Later stages of ethmoidal and sino-orbital mucormycosis that demonstrate proptosis and a blackened eschar over the ethmoidal area may require more extensive surgery and result in loss of vision in the involved eye. Ocular or optic nerve involvement is suggested by loss of vision. Posterior extension of the orbital infection may result in orbital apex syndrome with catastrophic infarction of the neurovascular structures within the optic canal (optic nerve and ophthalmic artery) and superior orbital fissure (cranial nerves III, IV, V1, VI), as well as veins draining the orbit into the cavernous sinus.

#### 6.1.3. Rhinocerebral Mucormycosis

Cavernous sinus thrombosis and other forms of rhinocerebral infection are advanced forms of mucormycosis that may be avoided if a diagnosis of an earlier stage of infection can be established. Cavernous sinus thrombosis typically arises from ethmoidal, sino-orbital, or sphenoidal mucormycosis. Initial infection of the cavernous sinus may reveal one or more cranial nerve palsies involving III, IV, V1, V2, or VI. The progression of infection may result in angioinvasion and thrombosis of the internal carotid artery with devastating cerebral infarction. A meticulous physical examination may detect these cranial nerve deficits before structural abnormalities are observed on CT scan or MRI diagnostic imaging.

Lateral extension of sphenoidal sinus mucormycosis into the contiguous cavernous sinuses also may lead to thrombosis and infarction of its neurovascular structures. Early MRI enhancement of a T2 signal within the sphenoidal sinus wall may portend further lateral extension into the cavernous sinuses.

Mucormycosis of the frontal sinus may also result in destruction of the calvarium with subsequent invasion of the dura, meninges, and frontal cortex. Clinical signs may be minimal, and the earliest manifestation may be the enhancement of an MRI T2 signal of the frontal bone or dural tissue.

### 6.2. Advanced Pulmonary Mucormcycosis

More advanced stages of pulmonary mucormycosis are reflected by the reverse halo sign, which is characterized by a mass consisting of a central area of ground glass opacification surrounded by a ring of dense consolidation [29,30]. The reverse halo sign and large nodular densities with halo signs signify angioinvasion, tissue infarction, and necrosis. As penetration of antifungal agents into such lesions is likely to be reduced and surgical resection may be the only means of eradicating pulmonary mucormycosis at this advanced stage. Pulmonary mucormycosis may be further complicated by invasion of the ribs, mediastinum, pericardium, myocardium, and diaphragm [31,32,33].

### 6.3. Advanced Cutaneous Mucormycosis with Musculoskeletal Infection

Lesions of advanced cutaneous mucormycosis are characterized as poorly healing ulcerations with indurated surrounding ischemic tissue, and progressive necrotic foci with hardened black eschars. More advanced stages of cutaneous mucormycosis may invade into deep subcutaneous tissues, muscle, tendon, and bone [41]. Repeated surgical resection of infected deep musculocutaneous tissue may be necessary as clinical estimates of the extent of infection at the time of surgery may be inaccurate.

### 6.4. Advanced Gastrointestinal Mucormycosis

Advanced mucormycosis of the gastrointestinal tract is complicated by the development of infarctions and perforations of the stomach and intestinal tract, resulting in combined bacterial and fungal peritonitis. Urgent surgical intervention is needed in order resect infarcted tissue as well as to debride infected peritoneal and tissue surfaces. Despite follow-up antifungal therapy and further surgery, mortality remains perilously high in advanced gastrointestinal mucormycosis.

### 6.5. Isolated Organ Mucormycosis

Isolated cerebral mucormycosis may be observed in immunocompromised patients with contaminated vascular catheters or in illicit intravenous drug users [1,42,43]. Target organ sites may be the kidney, brain, and heart. Mucormycotic peritonitis may develop during peritoneal dialysis. Early site-dependent diagnosis may lead to surgical intervention, antifungal therapy, and improved outcomes.

### 6.6. Disseminated Mucormycosis

As disseminated mucormycosis already represents an advanced stage of disease that carries an ominous prognosis, early detection and treatment may nonetheless stabilize infection and possible successful eradication. Since the brain is the most common site of dissemination, the unexplained development of focal neurological deficits in high-risk patients should include mucormycosis in the differential diagnosis, prompting urgent diagnostic imaging and biopsy of CNS lesions.

Disseminated mucormycosis can be rapidly fatal. Timely diagnosis is crucial to avoid delay of treatment. While awaiting confirmation of a diagnosis of mucormycosis, pre-emptive treatment should be initiated as promptly as possible [44].

## 7. Future Directions

As we look toward the future in meeting the challenges of mucormycosis, there are numerous promising advances in diagnostic tools and therapeutic interventions. These advanced diagnostic tools include, but are not limited to, host-based biomarkers, nucleic acid amplification platforms, matrix-assisted laser desorption/ionization–time of flight mass spectroscopy (MALDI-TOF MS), metagenomic next-generation sequencing of plasma cell-free DNA, and artificial-intelligence-guided interpretation of diagnostic imaging [45,46,47,48,49,50].

New therapeutic advances and novel molecular targets hold promise for new treatment approaches in management of patients suffering from mucormycosis. Among the promising antifungal small molecules are fosmanogepix (fungal GPI anchor inhibitor), SCY-247 (second-generation triterpenoid), and MAT2203 (oral nanoparticle amphotericin B) [51]. Among the new molecular targets which impact on virulence and innate host defenses are CotH, hypoxia-inducible factor-1α, and blockade of the PD-1/PD-L1 immune checkpoint pathway [20,52,53]. Host- and risk-based models may also help to increase clinical awareness for the early detection of mucormycosis [54]. Novel clinical trials designs may accelerate the translational development of innovative therapeutics to patients with rare invasive fungal diseases, such as mucormycosis [55].

While these encouraging advances should result in the improved care of our patients with mucormycosis, the important principles of recognition of host-based risk factors and meticulous detection of early clinical manifestations remain fundamental cornerstones to the diagnosis, treatment, and successful outcomes of this debilitating and frequently lethal disease.

## Figures and Tables

**Table 1:** Recognizing host factors for patients at risk for mucormycosis.

Metabolic disorders
Type I and type II diabetes mellitus (metabolic acidosis)
Primary aminoaciduria (metabolic acidosis)
**Hematological diseases**
Acute leukemia
Lymphoma
Severe aplastic anemia
Myelodysplasia
**Transplantation**
Hematopoietic cell transplantation
Solid organ transplantation
**Immunosuppressive therapies**
Corticosteroid therapy
CAR-T-cell therapy
Tyrosine kinase inhibitors (e.g., ibrutinib)
**Iron overload conditions**
Deferoxamine therapy
Multiple transfusions
**COVID-19**
Concomitant diabetes mellitus
Administration of corticosteroids
**Immunodeficiencies**
HIV/AIDS
Severe congenital neutropenia
Chronic granulomatous disease
**Previously immunocompetent patients**
Burns
Combat trauma
Civilian trauma
Severe weather victims (e.g., tornados, tsunamis)
Illicit intravenous drug use

**Table 2:** Host-based clinical manifestations of mucormycosis.

Underlying Risk for Mucormycosis	Predominant Anatomic Sites of Infection
Diabetes mellitus	Sinusitis; sino-orbital infection; rhinocerebral infection
Hematological malignancies, severe aplastic anemia, and myelodysplasia	Pulmonary infection; sinusitis; sino-orbital infection; rhinocerebral infection; cutaneous disease; disseminated infection
Hematopoietic cell transplantation	Pulmonary infection; sinusitis; sino-orbital infection; rhinocerebral infection; cutaneous disease; disseminated infection
Solid organ transplantation	Pulmonary infection; sinusitis; sino-orbital infection; rhinocerebral infection; cutaneous disease; disseminated infection
Prematurity	Gastrointestinal infection; cutaneous infections
Illicit injection drug use	CNS infection; musculoskeletal disease
Previously immunocompetent	Cutaneous and musculoskeletal diseases related to burns, trauma, and blast injury

**Table 3:** Recognizing early clinical manifestations of mucormycosis in high-risk patients.

Early Clinical Manifestations		Presence of Host Risk Factors (Table 1)		Possible Mucormycosis		Evaluation for Proven Mucormycosis
Diplopia (suggestive of entrapment of medial rectus muscle) Dysesthesia, pain, and/or erythema over the nasal bridge	**→**	**+**	**→**	Ethmoidal sinus mucormycosis	**→**	Ophthalmology consult CT scan of sinuses Otolaryngology consultation Nasal endoscopy if CT scan is consistent with ethmoidal sinus mucormycosis
Dysesthesia, pain, and/or erythema over the frontal area	**→**	**+**	**→**	Frontal sinus mucormycosis	**→**	CT scan of sinuses Otolaryngology consultation Neurosurgical consultation, depending upon findings of CT scan Biopsy of infected tissue
Dysesthesia, pain, and/or erythema over facial area Dental pain Unilateral palatal erythema and dysesthesia Nasal congestion Serosanguinous discharge	**→**	**+**	**→**	Maxillary sinus mucormycosis (sinusitis)	**→**	CT scan of sinuses Otolaryngology consult Nasal endoscopy and biopsy of suspicious lesions
Periorbital cellulitis (post-septal) Diplopia (suggestive of entrapment of medial rectus muscle)	**→**	**+**	**→**	Sino-orbital mucormycosis	**→**	CT scan of sinuses Ophthalmology consultation Otolaryngology consultation Nasal endoscopy and biopsy of suspicious lesions
Pain and headache localized over frontal sinus Upper nasal congestion Necrotic lesion on hard palate Soft-tissue swelling over frontal sinus	**→**	**+**	**→**	Frontal sinus mucormcycosis	**→**	CT scan of sinuses and brain Otolaryngology consultation Neurosurgical consultation, especially if dural enhancement is detected
Cough, pleuritic pain, dyspnea, and tachypnea	**→**	**+**	**→**	Pulmonary mucormycosis	**→**	Chest CT scan Pulmonary consultation BAL or transthoracic needle aspiration Serum/whole-blood PCR, cell-free NAAT, or whole-blood metagenomics
Non-healing wound Presence of black eschar	**→**	**+**	**→**	Primary cutaneous mucormycosis	**→**	Dermatology consultation General surgical consultation Biopsy of suspicious lesion
Direct penetrating injury	**→**	**+**	**→**	Musculoskeletal mucormycosis	**→**	General surgical consultation Biopsy of suspicious lesion
Localized abdominal pain Peritoneal signs	**→**	**+**	**→**	Gastrointestinal mucormycosis	**→**	Abdominal CT scan General surgical Laparotomy, if indicated

**Table 4:** Recognizing advanced clinical manifestations of mucormycosis in high-risk patients.

Clinical Disease	Advanced Clinical Manifestations
Ethmoidal sinus mucormycosis	Necrotic cutaneous lesions extending from invasion of the underlying ethmoid and nasal bones Diagnostic imaging demonstrating destruction of nasal and ethmoid bones
Frontal sinus mucormycosis	Pain and/or erythema over the frontal area Proptosis, ptosis Loss of EOM function
Maxillary sinus mucormycosis (sinusitis)	Necrotic lesion on hard palate Loss of dentition Extension into maxillofacial soft tissue Destruction of maxillary bone on diagnostic imaging
Sino-orbital mucormycosis	Proptosis, ptosis Loss of EOM function Chemosis Loss of vision
Rhinocerebral mucormycosis	Signs and symptoms of frontal, ethmoidal, or sphenoidal sinus infection with focal cranial nerve deficits Cavernous sinus thrombosis with injury to III, IV, V1, V2, and VI cranial nerves Cerebral hemispheric stroke caused by invasion of the internal carotid artery within the cavernous sinus
Pulmonary mucormycosis	Hemoptysis Reversed halo sign and infarcted lung on CT scan Invasion of ribs, mediastinum, and pericardium
Primary cutaneous mucormycosis	Presence of black eschar with extension
Musculoskeletal mucormycosis	Necrosis of muscle, tendon, and bone
Gastrointestinal mucormycosis	Infarction of stomach and intestines Perforation of stomach and intestines Mixed bacterial and mucormycotic peritonitis Mucormycotic invasion of abdominal and pelvic organs, as well as diaphragms and retroperitoneal structures

Abbreviations: EOM: extraocular muscle.
